# Mucopolysaccharidoses Differential Diagnosis by Mass Spectrometry-Based Analysis of Urine Free Glycosaminoglycans—A Diagnostic Prediction Model

**DOI:** 10.3390/biom13030532

**Published:** 2023-03-15

**Authors:** Francesca D’Avanzo, Alessandra Zanetti, Andrea Dardis, Maurizio Scarpa, Nicola Volpi, Francesco Gatto, Rosella Tomanin

**Affiliations:** 1 Laboratory of Diagnosis and Therapy of Lysosomal Disorders, Department of Women’s and Children’s Health, University of Padova, 35128 Padova, Italy; 2 Fondazione Istituto di Ricerca Pediatrica Città della Speranza, 35127 Padova, Italy; 3 Regional Coordinator Centre for Rare Diseases, University Hospital of Udine, 33100 Udine, Italy; 4 Department of Life Sciences, University of Modena and Reggio Emilia, 41125 Modena, Italy; 5 Department of Oncology—Pathology, Karolinska Institute, 171 77 Stockholm, Sweden

**Keywords:** mucopolysaccharidoses, diagnosis, mass spectrometry, glycosaminoglycans, urine, GAGome

## Abstract

Impaired glycosaminoglycans (GAGs) catabolism may lead to a cluster of rare metabolic and genetic disorders called mucopolysaccharidoses (MPSs). Each subtype is caused by the deficiency of one of the lysosomal hydrolases normally degrading GAGs. Affected tissues accumulate undegraded GAGs in cell lysosomes and in the extracellular matrix, thus leading to the MPS complex clinical phenotype. Although each MPS may present with recognizable signs and symptoms, these may often overlap between subtypes, rendering the diagnosis difficult and delayed. Here, we performed an exploratory analysis to develop a model that predicts MPS subtypes based on UHPLC-MS/MS measurement of a urine free GAG profile (or GAGome). We analyzed the GAGome of 78 subjects (38 MPS, 37 healthy and 3 with other MPS symptom-overlapping disorders) using a standardized kit in a central-blinded laboratory. We observed several MPS subtype-specific GAGome changes. We developed a multivariable penalized Lasso logistic regression model that attained 91.2% balanced accuracy to distinguish MPS type II vs. III vs. any other subtype vs. not MPS, with sensitivity and specificity ranging from 73.3% to 91.7% and from 98.4% to 100%, depending on the predicted subtype. In conclusion, the urine GAGome was revealed to be useful in accurately discriminating the different MPS subtypes with a single UHPLC-MS/MS run and could serve as a reliable diagnostic test for a more rapid MPS biochemical diagnosis.

## 1. Introduction

Glycosaminoglycans (GAGs), also called mucopolysaccharides, are complex linear polysaccharides constituted by sequences of repeating disaccharide units. They are covalently linked to proteins to form proteoglycans (PGs), which are located on the cell surface as well as in the pericellular regions and extracellular matrix, being involved in many cellular processes [1]. The main GAGs in humans are chondroitin sulfate (CS), dermatan sulfate (DS), heparan sulfate (HS), keratan sulfate (KS) and hyaluronic acid (HA). Except for HA, all the other ones are sulfated to a different extent and in different positions, thus creating peculiar sulfation motifs that confer GAGs various essential biological functions [2].

Impaired GAGs catabolism may lead to a series of disorders called mucopolysaccharidoses (MPSs). They are a cluster of 12 rare genetic metabolic diseases (including the recently identified MPS type X [3]), mainly affecting children and belonging to the wider group of lysosomal storage disorders (LSDs). Each of them is caused by the deficit of one of the lysosomal hydrolases normally degrading glycosaminoglycans within lysosomes [4]. These lysosomal hydrolases are coded by a series of housekeeping genes, expressed in most cell types, and each enzyme is known to selectively excise a single specific bond in one, sometimes more, glycosaminoglycans [4]. The prevalence of each MPS subtype varies based on ethnic background, and it may be influenced by founder effects [5]. On average, the registered MPS combined prevalence per 100,000 live births remains low as it ranges from 0.036 in Poland to 4.8 in Portugal, with the exception of Saudi Arabia, registering a prevalence of 16.9 affected subjects per 100,000 newborns [5].

Affected tissues accumulate undegraded GAGs in cell lysosomes and in the extracellular matrix, thus compromising their functions, and triggering the onset and progression of general inflammatory status, involving several body districts in most cases including the brain. All this leads to the known MPS clinical phenotype. Although MPS subtypes may present with recognizable, individual peculiarities, signs and symptoms often overlap [6], particularly in the early stages of the disease, making a timely, correct diagnosis more difficult. This is further aggravated by the possible overlap of symptoms with other metabolic or neurometabolic pediatric diseases. In addition, age at onset and disease progression may be very different from one patient to another; thus, patients may present at the onset with only one or a few symptoms of the disorder, enabling them to address a correct clinical diagnosis.

When the clinical examination raises suspicion of mucopolysaccharidosis, quantitative and/or qualitative urinary GAG assays are performed to estimate the extent and quality of GAG accumulation. Quantitative assays are usually colorimetric using 1,9-dimethylene blue (DMB); however, this method, although rapid and cheap, may give false positive or false negative results due to the presence of interfering substances in the samples [7,8,9]. Next, qualitative electrophoretic analysis can help with differentiating MPS subtypes based on the pattern of bands of the four main GAGs (CS, DS, HS and KS) [10], except for MPS I and MPS II, which remain undistinguishable, as well as the different forms of MPS III. However, in the last decade, an increasing number of laboratories have switched to assays based on mass spectrometry (MS) for differential diagnosis, given its superior accuracy, speed, specificity and sensitivity [11].

Recently, Tamburro and colleagues have developed and evaluated the analytical performance of ultra-high-performance liquid chromatography coupled with a triple-quadrupole tandem mass spectrometry (UHPLC-MS/MS)-based kit for the quantification of 17 free GAG disaccharides in urine samples. The kit measures eight CS disaccharides (including DS species, indistinguishable from CS), eight HS disaccharides and HA—collectively referred to as the free GAGome [12]. The kit showed acceptable linearity, selectivity and specificity, accuracy and precision and analyte stability in the quantification of 15 out of 17 GAG disaccharides.

In this paper, we report the results of a retrospective study using the above-mentioned UHPLC-MS/MS-based kit aiming at developing a prediction model to aid the differential diagnosis of mucopolysaccharidoses based on urine free GAGome measurements.

## 2. Materials and Methods

### 2.1. Study Population and Sample Collection

The study population consisted of 38 MPS patients (including MPS I, II, IIIA, IIIB, IVA, IVB and VI) and 37 healthy subjects. In addition, 3 patients affected by other disorders, 2 affected by mucolipidosis type II and one affected by spondylometaphyseal dysplasia-Kozlowski type (SMDK), whose symptoms may potentially overlap with those of MPSs, were included in the study.

MPS patients were included in the study according to the following inclusion criteria:

- Presenting with a definitive diagnosis of MPS of any sub-type confirmed by clinical, biochemical and genetic evaluation;
- Availability of a urine sample collected before the initiation of enzyme replacement therapy (ERT) for patients for whom the treatment was available;
- Availability of a urine sample obtained at the first available clinical evaluation for MPS IIIA, IIIB and IVB, for which no treatment is accessible.

Urine samples from single urinations or up to 24 h urine collections were used in the study. Samples were collected for diagnostic purposes. They were then stored at −20 °C until the analysis described here. All samples were anonymized prior to analysis.

### 2.2. GAGome Analysis by UHPLC-MS/MS

A GAGome analysis blinded to clinical data for each urine specimen was performed in singleton across 5 independent UHPLC-MS/MS runs using the MIRAM^®^ Free Glycosaminoglycan kit (Elypta AB, Solna, Sweden) at Lablytica Life Sciences AB (Uppsala, Sweden). This method was previously described [12,13]. A deviation from the kit protocol was introduced by performing a 4 h instead of a 1 h enzymatic digestion to achieve a complete HS digestion, which was expected to be significantly higher than physiological levels in this cohort. The GAGome included eight CS/DS disaccharides (hereafter reported as CS) (0S CS, 2S CS, 6S CS, 4S CS, 2S6S CS, 2S4S CS, 4S6S CS and TriS CS), eight HS (0S HS, 2S HS, 6S HS, NS HS, NS6S HS, NS2S HS, 2S6S HS and TriS HS) and one HA disaccharide. Measurements were expressed as semi-absolute concentrations in μg/mL, as well as relative concentrations (μg/μg_tot_ %) to the total for the corresponding GAG class (computed as the sum of the eight CS or HS disaccharide concentrations). Semi-absolute concentrations were then normalized by urine creatinine (measured as µg/mg creatinine), which was available for all patients since the standard-of-care prescribed re-sampling and recalibration for any sample initially presenting a creatinine value below the lower limit of quantification.

### 2.3. Statistical Analysis

We used a generalized linear model to correlate each GAGome feature (17 CS or HS or HA disaccharide semi-absolute concentrations, 16 CS or HS disaccharide relative concentrations, as well as total CS and HS concentrations) as a response variable to each MPS subtype vs. the control group as exploratory variables. The UHPLC-MS/MS run was added as a fixed-effect covariate to the model to adjust for run-to-run variability. The standardized change in a GAGome feature was tested against the null hypothesis of no change compared to the control groups using a two-sided t-test. Multiple hypothesis testing was accounted for using the Benjamini–Hochberg correction. The *p*-values < 0.05 were considered statistically significant. The statistical analysis was conducted using the *stats* package in R (4.2.2).

### 2.4. Development of Prediction Model for MPS Subtype Multiclassification

We developed a multinomial Lasso-penalized multivariable regression model using the urine free GAGome measurements as exploratory variables (consisting of 16 CS or HS semi-absolute concentrations as well as the 16 corresponding relative concentrations). We lumped the response variables into four classes: (1) not MPS (‘not MPS’), (2) MPS type II (‘MPS II’), (3) MPS type III (‘MPS III’) and (4) MPS of any type except II or III (‘MPS not II/III’). We performed 10-fold cross-validation and selected for the final model the regression coefficients at the penalty parameter λ that minimized the standard cross-validation error. The performance was evaluated on the same dataset in terms of balanced accuracy for the overall prediction across classes as well as in terms of sensitivity and specificity for three binary tests: MPS II vs. not, MPS III vs. not and MPS not II/III vs. not. The model was developed using the *glmnet* package (4.1-4) and evaluated using the *cvms* package (1.3.7), all in R (4.2.2).

## 3. Results

### 3.1. Characteristics of the Samples Analyzed

A retrospective single-center case-control study is carried out on 78 subjects, so grouped: 38 MPS patients across seven subtypes, 37 healthy subjects and 3 patients with non-MPS disorders. The last two groups are lumped into a single control group (Table 1). MPS vs. control patients have a mean age of 8.7 years vs. 6.2 years and have a balanced sex distribution (39.5% vs. 45.0% females). The MPS subtype distribution could be considered representative of the general population of potentially diagnosable MPS patients. No patients screened for this study have MPS IIIC, IIID or VII, which is expected given the rarity of such subtypes [14,15]. Neurological involvement was diagnosed in 52.6% of MPS patients.

### 3.2. Total Free CS, HS and HA Concentration

**Figure 1:**
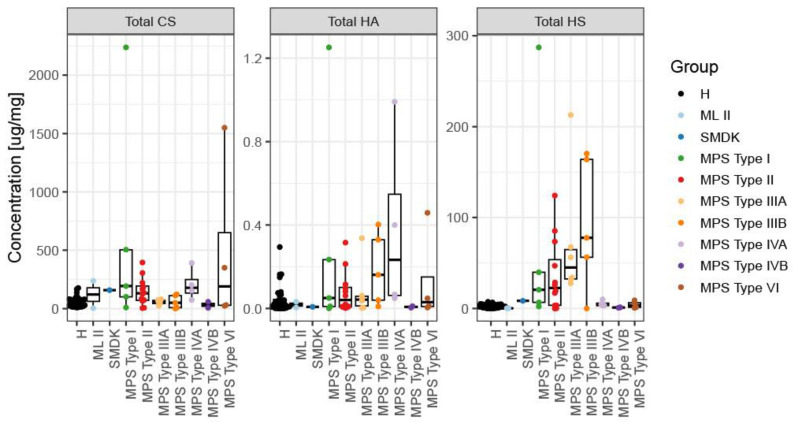
Total CS, HA and HS concentrations (µg/mg creatinine) measured in the different groups of MPS patients and controls (non-MPS patients—ML II, SMDK—as well as healthy subjects). SMDK: spondylometaphyseal dysplasia-Kozlowski type. ML II: mucolipidosis type II.

Creatinine-normalized values of total CS, HS and HA show an aberrant pattern in most MPS subtypes compared with controls, as expected (Figure 1, Table S1). Total HS shows a considerable increase in MPS IIIA and MPS IIIB, as well as in MPS I and MPS II, although to a minor extent. An increase in total CS and HA is primarily observed in MPS IVA. Total CS is also particularly increased in MPS VI, as expected. No increase of CS, HA and HS levels is registered in MPS IVB. This MPS subtype is known for commonly presenting normal levels of GAGs when evaluated with standard procedures, not including a specific evaluation of keratan sulfate, the only GAG accumulating in MPS IVB. The very elevated levels of CS, HA and HS detected in one of the MPS I samples analyzed are identified in the same patient, for whom a very high level of total urine GAGs/mg of creatinine is also measured by DMB analysis.

### 3.3. CS and HS Disaccharide Concentration and Composition

**Figure 2:**
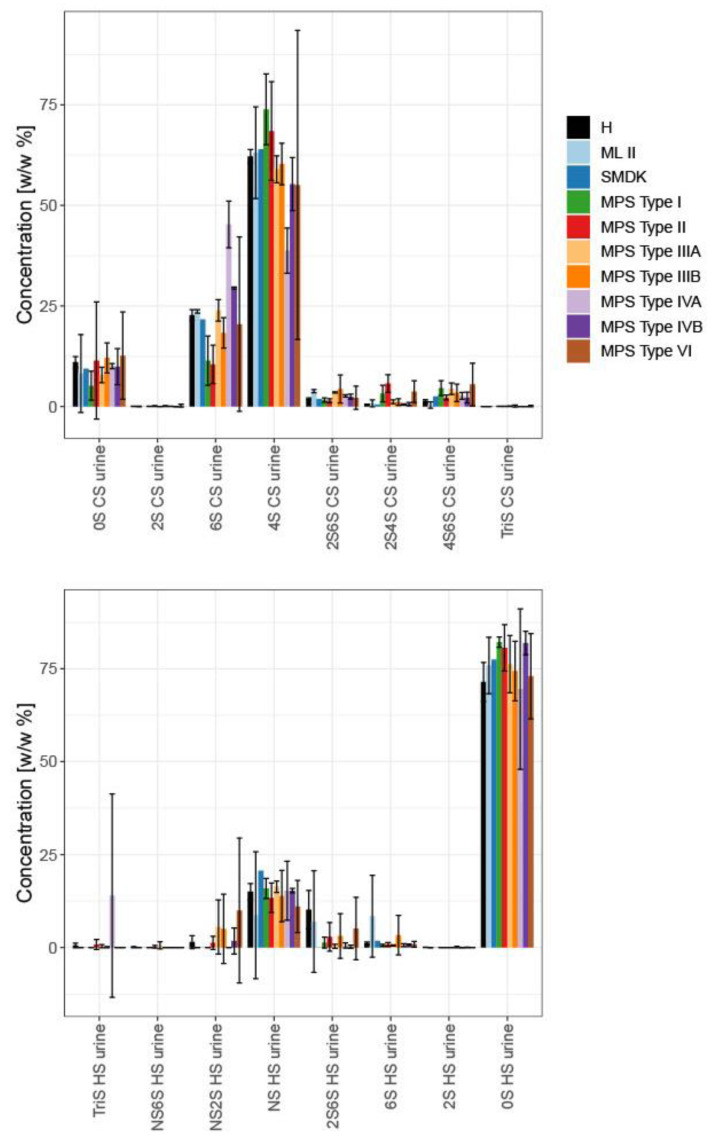
Relative concentration (in mass fraction %) of CS (upper panel) and HS (lower panel) disaccharides in the groups. SMDK: spondylometaphyseal dysplasia-Kozlowski type. ML II: mucolipidosis type II. See also Table S2.

The urine GAGome structure in MPS patients vs. controls is measured in terms of eight CS and eight HS disaccharide semi-absolute and relative concentrations (Figure 2 and Figure S1, Table S2). In CS, we observe an increasing trend for 4S CS concentration (µg/mg creatinine) in MPS I and MPS II and for 6S CS in MPS IVA. In terms of relative concentration (µg/ug %), we note a decrease in 6S CS % in MPS I, MPS II and MPS VI. As for HS, we observe an increased 0S HS and NS HS concentration (µg/mg creatinine) in MPS III and, to some extent, in MPS I and an increased 0S HS relative concentration (µg/ug %) in MPS I and MPS IVB.

### 3.4. Correlation between Urine GAGome Measurements and MPS Subtypes

**Figure 3:**
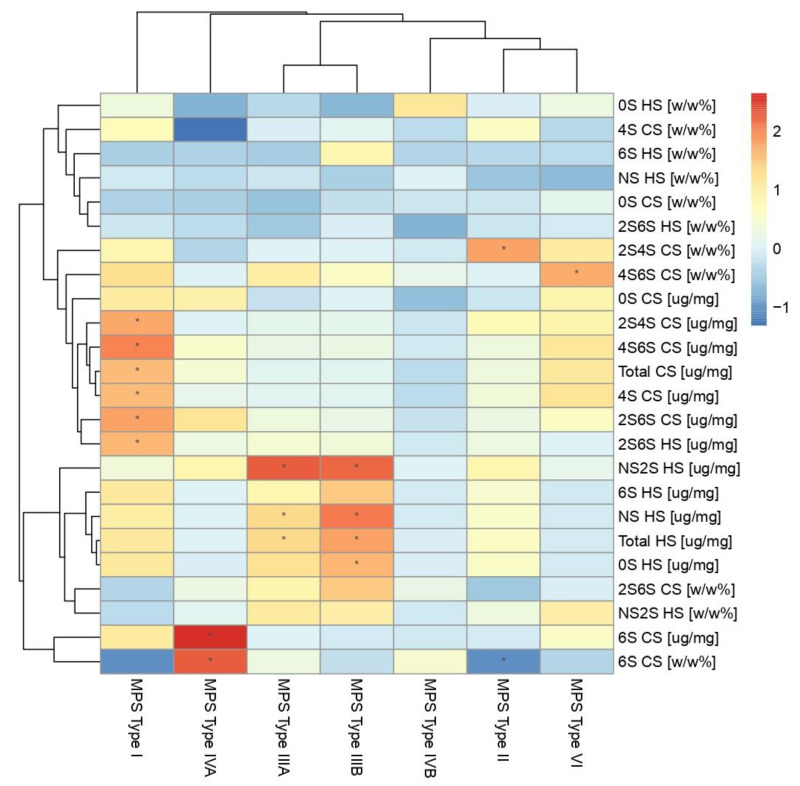
Heatmap of the GAGome profile across MPS subtypes compared to the control group. The color map is indicative of the standardized mean change for each GAGome measurement compared to the control group (red = increase and blue = decrease). Stars indicate statistically significant differences vs. the control group.

We investigated if changes in total or disaccharide GAGome measurements were significantly correlated with the different MPS subtypes compared to control subjects using a multivariable generalized linear model. The analysis reveals that many disaccharide-level changes are specifically correlated with different MPS subtypes (Figure 3). Notably, MPS II correlates with a specific increase in 2S4S CS % and a decrease in 6S CS %, MPS IIIA and B strongly correlate with increased NS HS, NS2S HS and total HS, with MPS IIIB also correlating with increased 0S HS. We observe an increase of 4S CS, disulfate CS, total CS and 2S6S HS only in MPS I. Conversely, we note a strong increase in 6S CS and 6S CS % only in MPS IVA and an increase of 4S6S CS % only in MPS VI. Consistently, in MPS type IVB, we found no significant correlations—as MPS IVB only accumulates keratan sulfate.

### 3.5. Development of a Prediction Model for the Differential Diagnosis of MPSs

**Figure 4:**
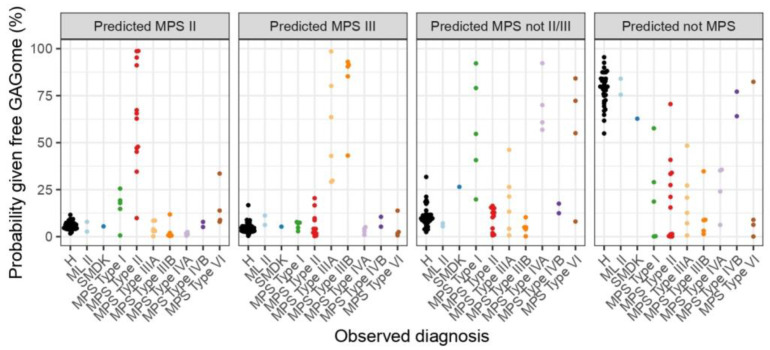
Prediction probability (in %) for each class as obtained by the prediction model developed for differential diagnosis of MPS subtypes, given the urine free GAGome measurements as inputs, vs. the observed diagnosis. SMDK: spondylometaphyseal dysplasia-Kozlowski type. ML II: mucolipidosis type II.

We developed a prediction model for the differential diagnosis of MPS subtypes using as sole input the urine free GAGome measured from a patient sample. Due to the low sample size for some MPS subtypes, we establish four prediction classes: (1) not MPS (‘not MPS’), (2) MPS type II (‘MPS II’), (3) MPS type III (‘MPS III’) and (4) MPS of any type except II or III (‘MPS not II/III’). The prediction model has 91.2% multiclass balanced accuracy with overall 86.7% sensitivity and 95.7% specificity. As shown in Figure 4, the probability predicted by the model for a patient to belong to a given class correlates well with the observed diagnosis. The prediction model allows the performance of three binary diagnostic tests at once using a single urine sample—for MPS type II, MPS type III or MPS not II/III—with sensitivity and specificity ranging from 73.3% to 91.7% and from 98.4% to 100%, respectively (Table 2).

## 4. Discussion

Due to the overlapping clinical signs and symptoms within the MPS group and with other metabolic disorders, and because of the high phenotypic variability among patients, timely diagnosis of mucopolysaccharidosis has long been difficult to achieve. Still today, in spite of the availability of several tools of analysis, in some cases the formulation of a diagnosis of MPS requires years, becoming a real odyssey for a few patients. Therefore, the availability of a sensitive test able to analyze urinary GAGs and to direct the MPS diagnostic pathway towards a specific subtype or to a group of MPSs could be very useful and would potentially lead to a shortening of the time to diagnosis. Moreover, a definite early diagnosis may precociously introduce patients to familiar genetic counseling as well as to the application of available therapeutic interventions, such as enzyme replacement therapy, so far developed for five MPS subtypes.

In recent years, several studies have tried to address this issue, mainly exploiting the advancements in MS and in the related chemistries able to depolymerize high molecular weight GAGs into smaller molecules. Saville et al. correctly diagnosed 10 MPS subtypes measuring urinary characteristic signature oligosaccharides with a terminal non-reducing end, reflecting the MPS-specific block in GAG catabolism [16]. The method identified all 93 MPS patients as belonging to one of ten MPS subtypes, from a total of 723 patients affected by metabolic disorders, thus showing 100% specificity and sensitivity. However, the lack of commercially available standards for each of the signature oligosaccharides is one of the limitations of this method. More recently, a similar procedure was used by the same group for the rapid identification of a chondroitin-sulfate disaccharide (HNAc-UA (1S)) as a diagnostic biomarker of MPS IVA [17]. In that study, the evaluation of the biomarker allowed the identification of a single MPS IVA patient within the over 2000 urine samples analyzed and to diagnostically redirect two patients who had been misdiagnosed for five years.

In the present study, we confirmed the aberrant pattern in the urine free GAGome of most MPS subtypes compared with controls. We observed elevated levels of total HS in MPS IIIA and IIIB patients, which is expected to be increased in the urine of these subjects. We observed the same elevation also in some MPS II and MPS I samples, which is consistent with the fact that both diseases are expected to accumulate HS and DS, although in different proportions. The concomitant CS analysis allowed us, however, to discriminate MPS I/II from MPS III A/B because CS concentrations were significantly different between these two groups of patients, hence allowing us to succeed in the differential diagnosis. On the other hand, MPS IVA, which is expected to show elevated levels of urinary CS due to the deficit of galactose 6-sulphatase, but not HS, showed HS concentrations similar to controls and a unique increase in one CS disaccharide, 6-O-sulfated CS. Finally, no increased GAG values were registered in MPS IVB samples, as expected, because in these subjects, the deficit of β-galactosidase causes altered levels of keratan sulfate [18], which was not measured in our analysis.

Based on the urine free GAGome profiles, we could develop a prediction model for a differential diagnosis of MPSs, reporting an overall 91.2% multiclass accuracy. The predictive model was able to correctly differentiate the four classes of samples as predicted ‘MPS II’, predicted ‘MPS III’, predicted ‘not MPS II nor III’ and predicted ‘not MPS’. Therefore, a single run combining the results obtained for the different GAG disaccharides measured by the kit should be able to accurately identify the MPS subtype class to which the patient belongs. The high sensitivity and specificity are promising as the model could aid clinical practice addressing the diagnostic workup towards an MPS subtype class and, consequently, towards a restricted number of confirmatory enzymatic assays, partially reducing the cost of testing as well as the time to diagnosis.

Nevertheless, there are limitations in this study that may preclude the generalizability of the prediction model results. First, even though the sample size was adequate considering the rarity of the disease and the strength of the associations underlying changes in the urine GAGome with the different MPS subtypes, a possible future implementation of the number of samples analyzed will help to better define or increase the model sensitivity. Second, the model was trained and tested in the same population, given the rarity of MPSs, incurring potential overfitting. Even if we took measures to reduce such risk, the model might have still been incorrectly calibrated. For example, populations from different locations may have different reference values of the urine GAGome compared with those observed in the controls of the present study. Altogether, we believe that although the model has shown high specificity and sensitivity in this study population, it should be tested and potentially recalibrated in external populations before its implementation in the clinical routine. Finally, the results obtained by the analysis of the two MPS IVB samples confirmed that, as expected, patients affected by this MPS subtype could not be correctly identified, strongly suggesting extending the UHPLC-MS/MS to include KS disaccharides identification. We believe that such improvement could further boost the diagnostic sensitivity of the prediction model.

In conclusion, the prediction model presented here was overall accurate in the differential diagnosis of MPS subtypes. Because all data analyzed here were obtained within a single UHPLC-MS/MS run, the model could be routinely used for screening applications to be conducted in reference diagnostic centers collecting and rapidly processing a significant number of urine samples—thus helping to reduce costs and manage the complexity of UHPLC-MS/MS instruments. This model could thus help shorten the time to diagnosis, positively allowing patients earlier access to available therapeutic options.

## Figures and Tables

**Table 1:** Study population baseline characteristics. Keys: H—healthy; MPS—mucopolysaccharidosis; SMDK—spondylometaphyseal dysplasia-Kozlowski type; ML II—mucolipidosis type II; and SD—standard deviation.

	Cases (MPS) (N = 38)	Controls (H/SMDK/ML II) (N = 40)	Overall (N = 78)
**Age**			
Mean (SD)	8.71 (8.37)	6.23 (5.57)	7.44 (7.14)
Median (Min, Max)	6.00 [0, 38.0]	4.00 [0, 24.0]	5.00 [0, 38.0]
**Sex**			
Female	15 (39.5%)	18 (45%)	33 (42.3%)
Male	23 (60.5%)	22 (55%)	45 (57.7%)
**Group**			
H	0 (0%)	37 (92.5%)	37 (47.4%)
ML II	0 (0%)	2 (5.0%)	2 (2.6%)
MPS I	5 (13.2%)	0 (0%)	5 (6.4%)
MPS II	12 (31.6%)	0 (0%)	12 (15.4%)
MPS IIIA	6 (15.8%)	0 (0%)	6 (7.7%)
MPS IIIB	5 (13.2%)	0 (0%)	5 (6.4%)
MPS IVA	4 (10.5%)	0 (0%)	4 (5.1%)
MPS IVB	2 (5.3%)	0 (0%)	2 (2.6%)
MPS VI	4 (10.5%)	0 (0%)	4 (5.1%)
SMDK	0 (0%)	1 (2.5%)	1 (1.3%)
**Neurological Involvement**			
Yes	20 (52.6%)	0 (0%)	20 (25.6%)
No	17 (44.7%)	0 (0%)	17 (21.8%)
Unknown	1 (2.6%)	0 (0%)	1 (1.3%)
Not applicable	0 (0%)	40 (100%)	40 (51.3%)
**Urine Creatinine (µg/mL)**			
Mean (SD)	64.7 (60.5)	70.4 (98.1)	67.6 (81.5)
Median [Min, Max]	45.5 [5.49, 364]	44.6 [3.00, 610]	44.6 [3.00, 610]

**Table 2:** Clinical performance of each binary test in the prediction model developed for differential diagnosis of MPS subtypes given urine free GAGome measurements as input. TP: true positive, TN: true negative, FP: false positive and FN: false negative.

Test Index	Test Positive	Test Negative	Sensitivity (95% CI)	Specificity (95% CI)	TP	TN	FP	FN
1	MPS II	Not MPS II	91.7% (61.5–99.8%)	100% (94.6–100%)	11	66	0	1
2	MPS III	Not MPS III	81.8% (48.2–97.7%)	100% (94.6–100%)	9	67	0	2
3	MPS not II/III	Not (MPS not II/III)	73.3% (44.9–92.2%)	98.4% (91.5–100%)	11	62	1	4

## Data Availability

The data presented in this study are available upon request from the corresponding author.

## Supplementary Materials

The following supporting information can be downloaded at: https://www.mdpi.com/article/10.3390/biom13030532/s1. Figure S1: Semi-absolute concentration (in ug/mg Creatinine) of CS (upper panel) and HS (bottom panel) disaccharides among groups. Keys: H—Healthy; MPS—mucopolysaccharidosis; SMDK—spondylometaphyseal dysplasia-Kozlowski type; ML II—mucolipidosis type II; SD—standard deviation, Table S1: Expected main GAG species accumulated by the different MPS subtypes across those included in the present analysis. CS and DS are not distinguishable by the kit tested, Table S2: Relative concentration (expressed in mass fraction %) of CS and HS disaccharides among groups. SMDK: spondylometaphyseal dysplasia-Kozlowski type, ML II: mucolipidosis type II.
