# Supplementary material for: Mucopolysaccharidoses Differential Diagnosis by Mass Spectrometry-Based Analysis of Urine Free Glycosaminoglycans—A Diagnostic Prediction Model
Source: Biomolecules. 2023 Mar 15;13(3):532. doi: 10.3390/biom13030532 (PMC10046358; doi:10.3390/biom13030532)
Supplement: Supplementary file 1 [file biomolecules-13-00532-s001.zip › biomolecules-2239553-supplementary.pdf]

Supplementary Material

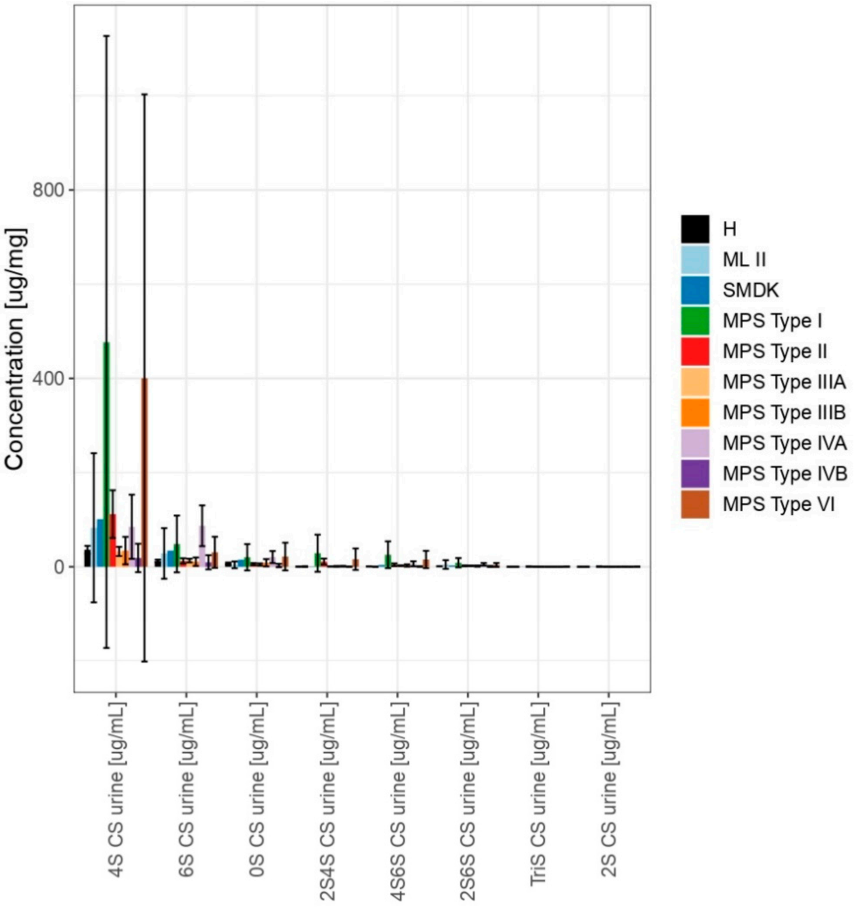

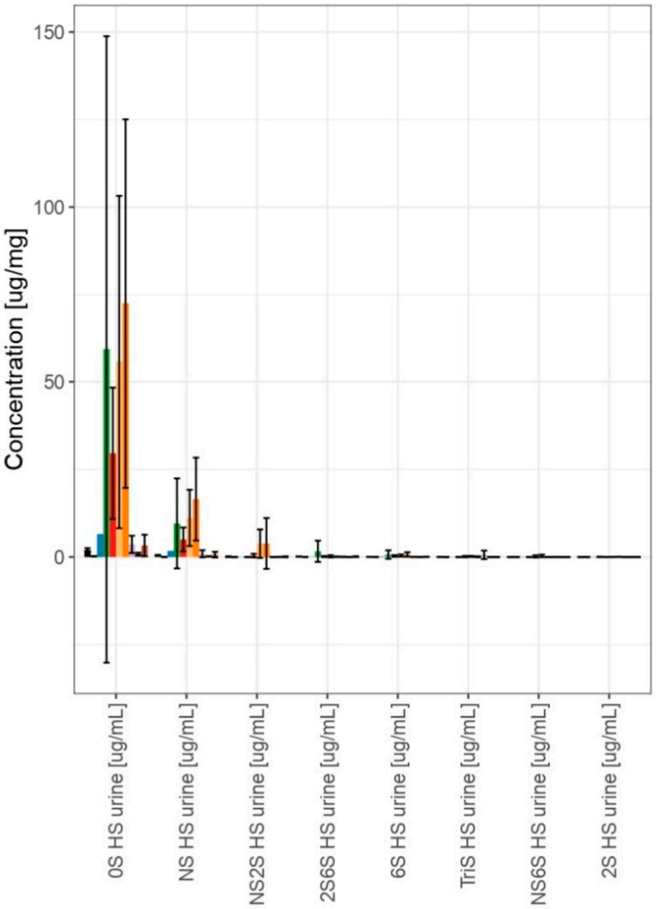

Figure S1: Semi-absolute concentration (in ug/mg Creatinine) CS (upper panel) and HS (bottom panel) disaccharides among groups. Keys: H - Healthy; MPS - mucopolysaccharidosis; SMDK - spondylometaphyseal dysplasia-Kozlowski type; ML II - mucolipidosis type II; SD - standard deviation.

**MPS subtype**	**CS**	**DS**	**HS**	**KS**
MPS I		✓	✓	
MPS II		✓	✓	
MPS IIIA			✓	
MPS IIIB			✓	
MPS IVA	✓			✓
MPS IVB				✓
MPS VI	✓	✓		

Table S1: Expected main GAG species accumulated by the different MPS subtypes across those included in the present analysis. CS and DS are not distinguishable by the kit tested.

Group	N	w/w %	0S CS	2S CS	6S CS	4S CS	2S6S CS	2S4S CS	4S6S CS	TriS CS
**Healthy**	37	Mean	11.03	0.05	22.73	62.21	1.94	0.53	1.47	0.03
		Standard Deviation	4.37	0.06	4.22	5.21	0.63	0.27	0.89	0.06
**ML II**	2	Mean	8.20	0.05	23.68	63.07	3.81	0.79	0.39	0.00
		Standard Deviation	7.00	0.05	0.30	8.22	0.29	0.61	0.55	0.00
**SMDK**	1	Mean	9.44	0.01	21.67	63.94	1.91	0.52	2.50	0.00
		Standard Deviation	N/A	N/A	N/A	N/A	N/A	N/A	N/A	N/A
**MPS Type I**	5	Mean	12.13	0.18	23.72	82.84	2.51	6.62	7.78	0.10
		Standard Deviation	4.12	0.05	6.97	10.03	0.61	2.33	2.11	0.05
**MPS Type II**	12	Mean	11.44	0.13	10.49	68.47	1.45	5.72	2.25	0.05
		Standard Deviation	25.71	0.23	8.46	21.73	0.88	3.92	1.09	0.08
**MPS Type IIIA**	6	Mean	7.87	0.04	23.91	58.98	3.51	1.19	4.42	0.08
		Standard Deviation	2.41	0.01	3.36	4.16	0.19	0.59	1.79	0.10
**MPS Type IIIB**	5	Mean	12.11	0.12	18.35	60.30	4.40	1.16	3.42	0.15
		Standard Deviation	4.27	0.12	4.31	5.88	3.97	0.93	2.49	0.31
**MPS Type IVA**	4	Mean	10.07	0.05	45.27	38.78	2.65	0.50	2.67	0.00
		Standard Deviation	0.64	0.03	5.95	5.75	0.28	0.14	0.86	0.01
**MPS Type IVB**	2	Mean	9.92	0.01	29.45	55.29	2.47	0.62	2.22	0.01
		Standard Deviation	3.22	0.02	0.18	4.74	0.42	0.29	0.98	0.01
**MPS Type VI**	4	Mean	12.65	0.22	20.49	55.09	2.20	3.73	5.50	0.13
		Standard Deviation	11.08	0.37	22.10	39.17	2.94	2.77	5.41	0.21

Group	N	w/w %	TriS HS	NS6S HS	NS2S HS	NS HS	2S6S HS	6S HS	2S HS	0S HS
**Healthy**	37	Mean	0.56	0.09	1.45	15.02	10.21	1.15	0.05	71.47
		Standard Deviation	1.85	0.51	5.47	6.81	15.97	1.07	0.06	16.25
**ML II**	2	Mean	0.00	0.00	0.00	8.71	6.98	8.40	0.02	75.90
		Standard Deviation	0.00	0.00	0.00	12.31	9.87	7.95	0.02	5.48
**SMDK**	1	Mean	0.00	0.00	0.00	20.66	0.02	1.80	0.02	77.49
		Standard Deviation	N/A	N/A	N/A	N/A	N/A	N/A	N/A	N/A
**MPS Type I**	5	Mean	0.00	0.00	0.00	19.43	3.48	1.10	0.06	83.84
		Standard Deviation	0.00	0.00	0.00	3.08	1.66	0.30	0.03	1.59
**MPS Type II**	12	Mean	0.83	0.21	1.23	13.38	2.87	0.84	0.03	80.61
		Standard Deviation	2.42	0.65	3.13	7.03	6.79	0.84	0.03	11.02
**MPS Type IIIA**	6	Mean	0.37	0.60	5.53	16.36	0.33	0.55	0.04	76.22
		Standard Deviation	0.51	1.22	9.07	1.90	0.69	0.17	0.00	9.64
**MPS Type IIIB**	5	Mean	0.08	0.00	5.03	13.86	3.12	3.34	0.15	74.41
		Standard Deviation	0.19	0.00	10.61	7.88	6.85	6.03	0.22	9.15
**MPS Type IVA**	4	Mean	13.97	0.00	0.00	15.29	0.56	0.64	0.02	69.52
		Standard Deviation	27.94	0.00	0.00	8.10	0.72	0.36	0.04	22.04
**MPS Type IVB**	2	Mean	0.00	0.00	1.79	15.29	0.20	0.76	0.02	81.94
		Standard Deviation	0.00	0.00	2.52	0.41	0.29	0.16	0.03	2.27
**MPS Type VI**	4	Mean	0.00	0.00	9.95	11.05	5.14	0.85	0.02	73.00
		Standard Deviation	0.00	0.00	19.90	7.19	8.58	0.78	0.01	11.73

Table S2: Relative concentration (expressed in mass fraction %) of CS and HS disaccharides among groups. SMDK: spondylometaphyseal dysplasia-Kozlowski type, ML II: mucolipidosis type II.
